# Evolution of disease modifying therapy clinical trial design and therapeutic endpoints for Parkinson's disease

**DOI:** 10.1177/1877718X261420002

**Published:** 2026-02-08

**Authors:** Tom Foltynie, Sonia Gandhi, Camille Carroll

**Affiliations:** 1 Department of Clinical & Movement Neurosciences, UCL Queen Square Institute of Neurology, London, UK; 2 The Francis Crick Institute, London, UK; 3 Translational and Clinical Research Institute Clinical Ageing Research Unit, Newcastle University, Newcastle, UK

**Keywords:** disease modification, clinical trials, drug development, trial design, outcome measures

## Abstract

The traditional approach of using double-blind, placebo controlled, parallel group trial designs has confirmed the efficacy of a large number of agents in relieving the symptoms of Parkinson's disease (PD) but has not, to date, led to the discovery of any disease-modifying treatments for PD. There are multiple potential reasons underlying the failure to find disease modifying approaches, which may in part relate to; inadequate understanding of PD pathophysiology and therefore inappropriate target selection; the possibility that even good candidate drugs may simply fail to reach and to ultimately engage with their putative targets at the required dose; and the significant heterogeneity of the disease both in terms of its pathophysiology and its motor and non-motor symptoms. PD also has some additional challenges that may be addressed by careful consideration of trial design. This includes; its generally slow rate of disease progression necessitating long follow-up times to identify evidence of disease slowing; lack of understanding regarding the optimal stage of disease that might be most amenable to intervention; as well as lack of consensus regarding which outcome measures best capture patient relevant disease progression, and which biomarkers might consistently and objectively provide the earliest indication of disease progression. In this review we will discuss these issues and potential approaches that may help in the evolution of clinical trial design and thus ultimately provide a pathway to increase the likelihood of successful identification of disease-modifying treatments for Parkinson's disease.

## Plain language summary

We have not yet discovered any medications or other interventions that slow down the progression of Parkinson's disease. This may be partly because we have tested the wrong medications in the wrong people but may also be due to the way in which we design and carry out clinical trials. In this article, we will discuss these issues and highlight the strategies that are being set up to try and improve our chances of success.

## Slowing down a heterogeneous disease

The clinical diagnosis of PD encompasses people with an array of different symptoms and signs who may all meet diagnostic criteria derived with reasonable sensitivity and specificity to predict the pathological definition of PD.^1^ The presence of alpha synuclein containing Lewy bodies are the cardinal feature of PD, and (while acknowledging the frequent co-existence of other pathological processes), great progress has been made in identifying the presence of abnormal forms of alpha synuclein during life as a means of improving the diagnostic process.

The presence of pathological alpha synuclein can be identified using seed amplification assay (SAA) techniques.^2^ This currently requires an invasive procedure to collect either CSF or skin (although the ability to detect pathological forms of alpha synuclein with peripheral blood samples may be moving closer.^3,4^) The PPMI study has shown that the majority of people with clinically diagnosed PD are positive for CSF alpha synuclein SAA, especially if they have olfactory loss. In contrast, the percentage of individuals with LRRK2 positive PD who are positive for the alpha synuclein SAA is far lower.^5^ The use of this test as a trial inclusion criterion would certainly help exclude people with non-PD tremors or atypical forms of parkinsonism from trials of treatments targeted towards alpha synuclein pathology. While CSF or skin sample collection may not be uniformly acceptable to all trial participants, routine collection of peripheral samples which should (hopefully soon) allow presence/quantification of alpha synuclein SAA should become imperative for trials targeted towards the amelioration of alpha synuclein related disorders.

A proposal to redefine PD as ‘neuronal synuclein disease” has embraced the importance of the SAA diagnostic biomarker,^6^ although is not free of controversy.^7^ The relationship between alpha synuclein SAA positivity and Lewy body pathology observed immunohistochemically among LRRK2 mutation carriers is less consistent^8,9^ and the concept of neuronal synuclein disease is likely to evolve further. There is also clear recognition that this same biomarker can be associated with a different phenotype- Lewy body dementia (DLB), and the consequence of revolutionising the conceptual definition of PD in this way, is that PD and DLB become considered together, which potentially may overlook factors relevant to how this specific aspect of the pathophysiology impacts on clinical phenotype and clinical progression. In parallel, there have been multiple attempts to further identify subgroups of PD which might more closely share an underlying pathophysiology. These have been based on clinical phenotype,^10^ Mendelian genetics,^11^ polygenic risk scores,^12^ cluster analysis or machine learning approaches to multimodal datasets^13^ but all have been subject to criticism.^14^

From the perspective of disease-modifying trials, the best approach to subclassification must be with respect to the underlying pathophysiological processes driving progression, and the intervention being tested. For example, individuals with GBA1 mutations are certainly best suited towards GBA1 based gene therapy approaches^15^ which might restore the lost enzymatic activity seen in GBA1 disease, and any consequent abnormalities in alpha synuclein aggregation, mitochondrial dysfunction and neuroinflammation would (presumably) autocorrect if the primary driver of neurodegeneration is alleviated before irreversible consequences can occur. Similarly, carriers of LRRK2 mutations are intuitively also the most likely to benefit from LRRK2 inhibition^16^ However this may be irrespective of SAA positivity^17^ (given that the same LRRK2 mutations can lead to non-PD pathologies,^18^). It is also possible that bidirectional processes exists between LRRK2 and downstream PD pathophysiology, such that LRRK2 inhibitors may impact beneficially even as compensation for other causes/triggers of PD. Existing trials evaluating LRRK2 inhibitor approaches are evaluating the impact of these drugs on both LRRK2 positive and negative participants,^19^ and there is an argument in support of genotyping all PD trial participants ahead of recruitment into any clinical trial of a disease modifying candidate, to allow enrichment of specific genetic groups or counterbalancing across active/placebo treatment arms.

Individuals with these types of single-gene disorders as the primary cause of their Parkinson's are however, the minority of individuals with the disease. While there are undoubtedly genetic influences on the evolution of disease among “sporadic” patients, other contributory triggers such as peripheral or central neuroinflammation,^20^ the role of sex hormones,^21^ the role of insulin resistance,^22^ exposure to environmental toxins,^23^ physical trauma or psychological stresses,^24^ leading to multiple aspects of neuronal dysfunction may account for a larger population attributable risk fraction of the disease. The primary causes of neurodegeneration may also not be the same as those factors which influence its subsequent rate of progression.^25^ In other words, even if not the primary triggers for neurodegeneration, intervening in the evolution of downstream deleterious processes may also have potential major impact in effective disease slowing.

A further consideration is the disease stage, beyond which intervention may be too late. Intervening early is intuitively more likely to be helpful before irreversible neurodegeneration has occurred. This approach has recently evolved from recruiting early untreated PD patients, to recruitment of individuals in the prodromal period, targeting people who have only developed non-motor symptoms such as REM sleep behaviour disorder, or olfactory loss. The parallel option is to recruit those individuals known to carry a PD risk gene but not yet manifesting any symptoms or signs of motor PD.

The counter argument however, relates to the very large prevalent population of PD who have already manifested motor symptoms and signs, but have many years or even decades before they develop the most troublesome dopa refractory gait and balance difficulties, speech and swallowing issues or dementia. Identifying whether any disease modifying approach might have beneficial effects through prevention of these disabling complications in this large prevalent population will remain a high priority.

## Treatment selection

Beyond development of therapies for single gene causes of PD, there are also emerging insights into druggable PD pathophysiological processes, which could plausibly have a disease modifying role, relevant to larger numbers of PD patients.^26^ Some candidate disease modifying interventions may intervene at a specific trigger point in the neurodegenerative pathway and have relevance for only a small number of people with PD. Other candidate interventions may act at points of convergence in the pathophysiological pathway e.g., by allowing rescue of a proinflammatory, bioenergetic or protein aggregation cycle that may be of benefit to a much broader population.

Undoubtedly certain compounds are selected for clinical trial evaluation based purely on commercial reasons, which will themselves be influenced by the size of the eventual market. In an academic setting, it is even more important to operationalise a process for selecting the highest priority compounds, based on the breadth and the strength of the existing evidence and thus the likelihood for eventual success.^27^

Drug repurposing of compounds which already have a license for human use reduces the risk of failure due to unrecognised safety concerns or tolerability issues. In the evaluation of repurposed drugs, there may be existing epidemiological data regarding the incidence of PD among people exposed to such candidate compounds for their existing indications. For example, a careful analysis of the UK THIN database revealed a markedly lower risk of PD among type 2 diabetes mellitus (T2DM) patients exposed to GLP1 receptor agonists, compared to T2DM patients treated with other glucose lowering agents.^28^ The processes influencing PD risk may however differ between people with and without T2DM therefore it cannot automatically be concluded that these drugs may be relevant for disease slowing in a PD population without T2DM. Furthermore, reducing the incidence of disease does not inevitably equate to an influence on slowing the progression of disease once the process has already become established.

Preclinical data, using in-vitro cell models, alongside in-vivo models of PD can provide very useful insights into the ability of a candidate compound to engage relevant PD targets. Over-reliance on acute toxin-induced models of PD may have contributed to previous failures of interventions when taken into clinical trials. While no in-vivo model of PD accurately recapitulates the human form of the disease,^29^ the choice of in-vivo model to judge a compound's potential, depends on the putative mechanism of action of the candidate drug, i.e., a drug considered to reduce alpha synuclein aggregation requires testing in a model in which alpha synuclein aggregation can be accurately quantified.^30^ In contrast, an agent which has a direct effect on mitochondrial bioenergetics might require parallel assessment in a mitochondrial model of PD.^31^ Notwithstanding this recommendation, we are also highly aware of the impact of alpha synuclein pathology on mitochondrial integrity and function,^32^ as well as the potential role of mitophagy induction in synuclein models of disease.^33^

Beyond assessing the preclinical credentials of a compound, establishing proof of concept of disease modification in a clinical setting is vital. There is a well-established pathway for the assessment of new “first in human” compounds to assess safety and tolerability in single then multiple ascending dose studies. These steps can be skipped for agents already licensed for use in humans, however the issue of dose finding to assess whether the drug accesses and properly engages its target remains just as important. This aspect of drug evaluation can itself be optimised and trial platforms aligned specifically at dose finding and evaluation of target engagement are emerging, which can provide the definitive information necessary prior to selecting treatments for formal efficacy evaluation.^34^

Attempts to reach consensus regarding the treatment prioritisation process have been sought for a number of years.^35,36^ These are inevitably influenced by shifting evidence over time, alongside the unpredictable nature of trial funding and the overall objectives of the trial. The extensive and ongoing work performed by the International Linked Clinical trials (iLCT) team^35^ is of enormous value and has been carefully integrated into treatment selection processes for disease modifying trials. Clinical evidence must carry the greatest weight compared to preclinical data, and logistical issues such as drug cost, tablet size, dosing regime become of immediate relevance, requiring discussion with potential trial participants, prior to final decisions being taken.

## Setting up a clinical trials machine- the platform approach

The number of drug candidates that already have existing preclinical or early clinical data to support their proof of concept as disease modifying drugs, demands that an efficient approach is developed to prove/disprove their real world potential. A number of complex innovative trial designs have been developed largely in the cancer trial field including Basket trials (testing one drug in multiple different groups/subgroups), Umbrella trials (testing multiple different agents within the same group), and Platform trials.^37^ Platform trials generally involve multiple agents being tested contemporaneously in different patients randomised to receive one of multiple active treatments, and their subsequent outcomes compared to a shared placebo arm. Interim analyses identify whether agents show any sign of activity, and if not, they can then be replaced by new candidate drugs. This type of design establishes an Infrastructure, i.e., maintains relevant teams of people who have the training and skills to assess/monitor trial participants over the longer term.

Fundamentally it can be appreciated that the likelihood of successful identification of disease-modifying compounds will increase simply by increasing the number of strong treatment candidates that proceed to formal clinical trial evaluation. For this reason, multi-arm platform trials have already been embraced in PD.^38^ A team in Australia have recruited to a randomised Phase 2 trial evaluating patients allocated for treatment with Albuterol (a beta adrenoceptor agonist which may reduce alpha synuclein gene expression), Alogliptin (a dipeptidyl peptidase 4 inhibitor which elevates Glucagon-like peptide 1 levels) .and Nilvadipine (a brain penetrant dihydropyridine calcium channel blocker) compared to placebo and including genotyping of all participants to explore pharmacogenomic effects.^39^ The Path to Prevention trial (P2P) is another multi-arm trial in set up, which will be conducted in people with ‘prodromal PD’, who have participated in the PPMI project and identified as having hyposmia or REM sleep behaviour disorder, and who have abnormal DATSCAN imaging at baseline. They will be randomly allocated to active drug interventions or placebo and followed longitudinally to identify any difference in the rate of progression of DATSCAN imaging or the emergence of motor signs of PD, or cognitive impairment. Intuitively it may be easier to slow the progression of PD pathology at an earlier point in its evolution, and this is the approach being taken by the P2P platform trial. In this population the SAA may have additional utility in determining when the pathophysiological processes of PD have started.

**Figure 1.**
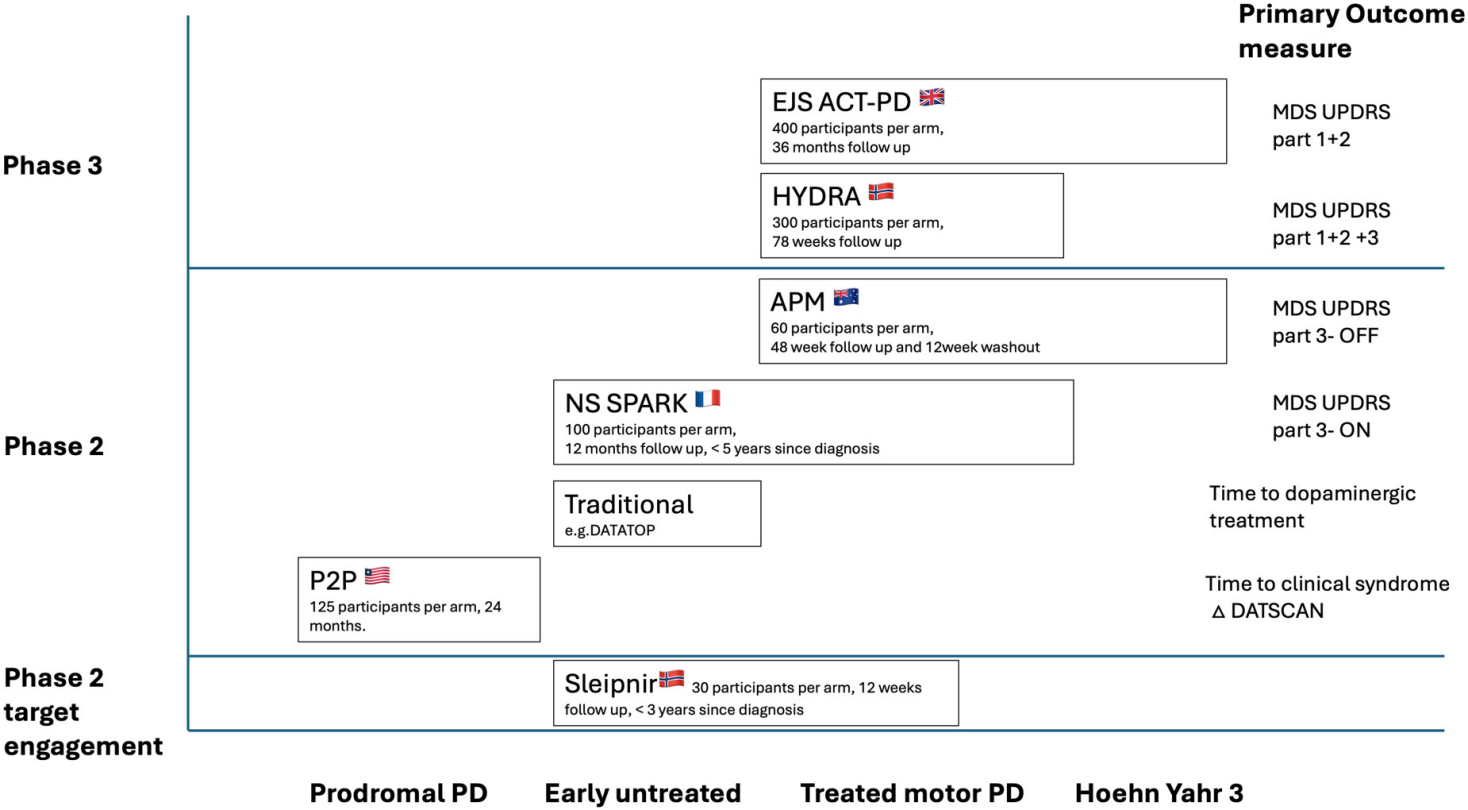
Summary of the major Platform trials either in set up or already recruiting in Parkinson's disease. Modified from Fabbri et al. Advantages and Challenges of Platform Trials for Disease Modifying Therapies in Parkinson's Disease. Mov Disord. 2024 Sep;39(9):1468–1477.

These trials (Figure 1) are very large, long term (and thus expensive) projects and require major investment from funding bodies, or carefully defined relationships with pharma companies to set-up and maintain. Long term funding inevitably requires careful navigation of commercial issues, and intellectual property rights, but these are potentially surmountable in the face of reduced overall costs and time required to reach definitive decisions regarding the efficacy of candidate drugs. In the planning of these trials, it is fundamentally important that the views of people with PD and their carers are fully incorporated at the design stage, to ensure that eventual trial results are directly relevant to their priorities.

## The first MAMS trial in Parkinson's disease-EJS ACT-PD

**Figure 2.**
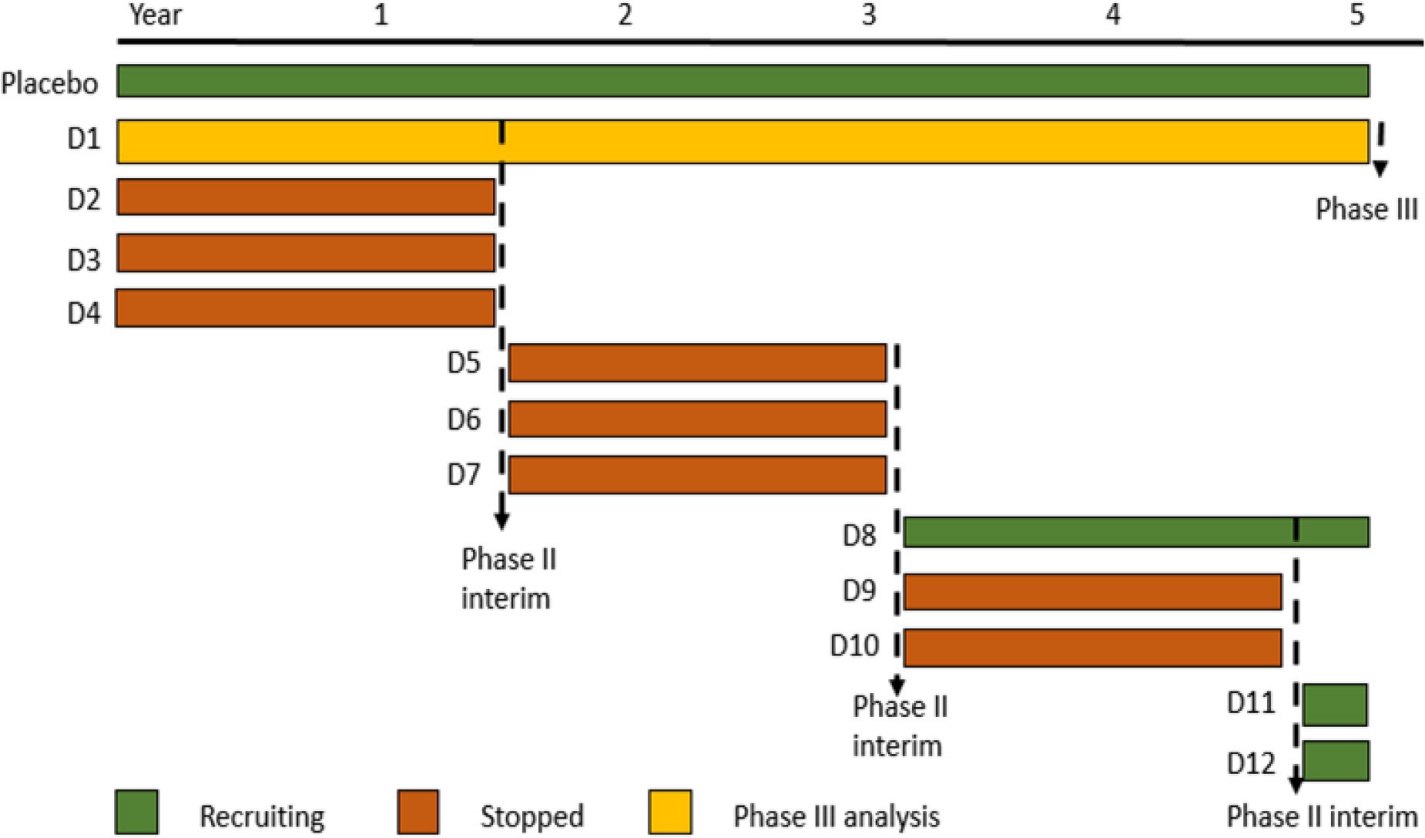
A schematic representation of a multi-arm, multi-stage platform trial. D1, D2 refer to drug arms 1, 2 etc. After a defined interval, an interim analysis is undertaken and only agents passing a predefined threshold (e.g., D1) will continue follow up and recruitment. D2, D3, D4 all fail at this threshold and are replaced by the next candidate interventions D5, D6 and D7. In this example D1, passes all interim assessments and undergoes formal efficacy analysis (Phase III equivalent) against the shared placebo arm.

A further efficiency that can be introduced into a Multi-Arm, platform trial is to include a Multi-Stage analysis approach- the MAMS design (Figure 2). In the context of a multi-arm trial, interim analyses can identify agents which show no sign of activity, which can then be replaced by new candidate drugs, whereas those agents surviving interim analyses can continue recruitment and follow up until sufficient data have accumulated to formally assess their efficacy and inform on licensing decisions. The advantage of this approach is that a single trial platform can produce multiple relevant results; fewer patients are randomised to placebo; and the design avoids the need for pausing in between a Phase 2 (interim) stage and final Phase 3 (efficacy) stage thus speeding up the process. MAMS Platform trials have been particularly efficient in the identification of effective treatments for prostate cancer,^40^ and are now also established to help in the identification of effective treatments for ALS,^41^ Multiple Sclerosis.,^42^ and most recently in PD.^43^

Using a MAMS type of trial design can also increase the efficiency of drug evaluation in Parkinson's disease but comes with additional challenges.^44,45^ To enable robust, patient-centric decision-making regarding the design of a MAMS platform trial for PD, the Edmond J Safra Foundation supported the EJS ACT-PD (Accelerating Clinical Trials in Parkinson's disease) initiative which allowed the creation of 6 working groups to address all relevant issues and ensure all the relevant stakeholders were involved in the creation of the EJS ACT-PD trial protocol.^43^ Given the huge resources required for a MAMS platform trial, it was essential that the views and priorities of people with PD, were properly incorporated into the trial design. Integrating the PPI voice into all trial decisions has been fundamental to all aspects of the planning of the EJS ACT-PD MAMS Platform trial.^46^

From the outset, the EJS ACT-PD trial protocol required careful consideration that, fortunately, the rate of progression of PD is usually quite slow, although still leads to the relentless accumulation of disability. For this reason, using a hard endpoint such as mortality or metastasis (typical of the cancer MAMS trials) would be inappropriate. Given the absence of any validated biomarker of PD progression, the slow clinical course and therefore necessary long term follow up, increasing the efficiency of drug testing by avoiding pauses in recruitment and follow up, for those agents that pass interim thresholds for activity, keeps the duration of the clinical trial process for successful treatments to demonstrate efficacy as short as possible.

For a MAMS platform in PD to be successful, the issues of patient heterogeneity and the processes of treatment selection need to be aligned. In the first instance, prioritising interventions that are likely to be relevant to the majority of PD patients allows broader inclusion criteria and faster recruitment rates and will ultimately have greater impact on larger numbers of people.

## Patient selection in EJS ACT-PD

The population of patients selected for inclusion in a disease modifying trial need to be representative of those individuals who would eventually hope to be prescribed the medication. The focus in EJS ACT-PD is those individuals with clinically manifest motor symptoms and signs of PD, in whom there is still clearly scope for clinical worsening that might potentially be slowed/avoided through exposure to an effective drug.

The impact of prescription of dopaminergic medication has had to be carefully considered. Recruitment restricted to individuals who are not yet started on L-dopa replacement, avoids the influence of dopaminergic treatment on disease severity, but greatly limits the duration of follow up, before the introduction of dopaminergic treatment becomes clinically essential. The subsequent impact of starting dopamine replacement therapy on the trial outcome measures will likely outweigh any impact of the study drug on disease progression. The alternative approach, strongly supported by the views of the EJS ACT-PD PPIE group, is to recruit participants after starting on L-dopa replacement and then evaluating disease progression in the dopamine treated state over the longer term.^47^

There are many indisputable reasons underlying the importance of being inclusive in PD research, which have been recognised but largely ignored for many years.^48^ Inclusivity brings additional challenges from the perspective of patient heterogeneity but addressing the heterogeneity of PD should be considered on the basis of biochemical or genetic data rather than on age, sex, socioeconomic status or ethnicity. While EJS ACT-PD aims to be broad in its inclusion criteria, all participants will have baseline biomarker data collected with respect to genotyping, evidence of dysregulated lysosomal, mitochondrial or neuroinflammatory pathways in peripheral blood samples, and/or propensity to alpha synuclein aggregation seen in peripheral extracellular vesicles,^4^ that (separately or in combination) will allow subgroups to be defined.

Once fully established, EJS ACT-PD will also be able to introduce greater precision medicine approaches and allow subgroup analyses of a-priori defined groups, according to the mechanisms of action of the included interventions, as well as exploration of interventions which are more targeted among subgroups of patients, as has been successfully achieved in the Stampede trial.^49^

## Outcome measurement in EJS ACT-PD

Even in the absence of a disease progression biomarker, there has yet to be agreement on the optimal way of measuring PD progression.^50^ The traditional means of evaluating disease severity in PD generally relies on the Movement Disorders Society's Unified Parkinson's disease rating scale (MDS UPDRS). Part 1 of the scale captures non-motor symptoms, Part 2 captures the impact of motor symptoms on activities of daily living, Part 3 is a clinician's assessment of the motor severity of PD and Part 4 quantifies the extent of motor complications of the disease i.e., fluctuations and dyskinesias.^51^ Table 1 summarises the most important outcome measures relevant to PD disease modifying trial design.

**Table 1.** Summary of the key measures of importance in clinical trials of disease modifying interventions in Parkinson's disease.

	Diagnostic/Inclusion criteria	Measuring target engagement	Measuring disease progression
**Clinical**			
Clinician reported Patient reported	MDS Diagnostic criteria		MDS UPDRS part 3 MDS UPDRS part 12,4 Motor/Non-motor Milestones
**Wet Biomarker**			
Alpha synuclein Neuroinflammatory Lysosomal dysfunction Mitochondrial Synaptic Dopamine metabolites Axonal	SAA Total alpha syn p129 alpha syn Oligomeric alpha syn GCase activity Glucosylceramide Glucosylsphingosine PGC1α Neurogranin HVA, DOPAC	*Quantitative SAA * Total alpha syn p129 alpha syn Oligomeric alpha syn Interleukins Chemokines GCase activity Glucosylceramide Glucosylsphingosine NfL	*Quantitative SAA*
**Digital**			
			Passive wearables Active device based Apps Motion detection devices
**Imaging**			
MRI/ PET/SPECT	Structural MRI Dopamine SPECT/PET FDG PET *Alpha synuclein PET*	MR Spectroscopy Microglial PET *Alpha synuclein PET*	*Quantitative MRI * Dopamine SPECT/PET FDG PET *Alpha synuclein PET*

Italicised items are still in development.

Formal consultation with PD patients and their care partners clearly emphasised that the non-motor symptoms of PD are just as relevant to patients as the motor symptoms, (possibly influenced by the existence of dopaminergic replacement therapies which are often more effective for the motor symptoms of the disease). The functional impact of motor symptoms is also far more relevant to individuals living with the condition than the clinician's assessment of their slowness, stiffness, tremor, gait and balance. This aligns with regulatory advice that outcome measures in phase 3 trial reflect change that is clinically meaningful to patients with the illness.^52^ Furthermore, the major benefits of dopamine replacement strategies mean that the day to day experiences of patients with PD is in the dopamine-treated state, and it is the emergence/progression of the dopa-refractory symptoms that remains the major unmet need in PD. Insisting that participants attend trial visits in the Off dopaminergic medication state is inconvenient and potentially distressing to patients, and not additionally insightful on the rate of progression of dopa-refractory symptoms and signs.^47^

As a result, the combination of MDS UPDRS part 1 & 2 summed score has been chosen as the primary outcome of the EJS ACT-PD trial. This combination has clinometric validity and has been shown to change consistently over time using longitudinal data collected from a range of trials and studies (CPP). The use of a Patient reported outcome measure (PROM) does however pose the challenge of placebo effects and the critical importance of adequate blinding to treatment allocation. In parallel, there is substantial interest in the development of “Milestone-based approaches” to capture patient relevant disease progression.^53,54^ The optimal selection of which milestones to use, the specific details that define that a milestone has been reached and the methods for analysis of this type of approach still need to be carefully worked out.

## Placebo effects in platform trials

Placebo effects in PD are well recognised and may be variable according to the type of intervention and its mode of administration e.g., oral, vs injectable vs neurosurgical approaches.^55^ Previous candidate PD disease modifying interventions have all been associated with placebo effects with perhaps the greatest effects being seen in trials using the more invasive procedures. Expectation of benefit is a major determinant of the magnitude of the placebo effect,^56^ but how long placebo effects may last is uncertain.^57^ In theory the magnitude of a placebo effect might also be variable depending on the size, colour or frequency of administration of the intervention, even when comparing oral tablet dosing. To address this, EJS ACT-PD has ensured that the first treatment arms and placebo arm have identical size, shape, and colour of tablets all given once daily.

A further suggestion to reduce the impact of placebo responses is to administer placebo to all participants during a run in period prior to randomisation, and excluding those with a placebo response.^58^ This has however been shown to not necessarily be an effective strategy in PD.^57^ A further caveat in the interpretation of placebo effects relates to the reliability of an individuals’ baseline assessment. When first attending a trial appointment, an individual may be more stressed due to unfamiliarity with the location, team and trepidation about what the trial involves. Subsequent visits post intervention may be far more familiar, comfortable and less stressful, hence improvements in disease ratings may be driven by factors other than placebo effects/benefits of the intervention. To overcome this type of influence, EJS ACT-PD will incorporate repeat assessments of disease severity at both screening and baseline visits including both clinician rated and patient reported outcome measures.

The P2P trial will use DATSCAN progression as an objective measure of disease progression which will be free form observer bias and placebo effects, however a decision regarding whether an individual has developed clinically manifest evidence of motor PD or cognitive impairment separately from healthy aging may also be vulnerable to placebo effects, emphasising the need for better objective biomarkers of disease progression.

## Validating biomarkers for use in disease modifying trials

While a clear reflection of what matters most to patients, the rate of change of PROMs necessitates very long follow up, which is inefficient and expensive. Developing alternative objective measures of clinical disease progression is a very high priority. There are a large number of digital devices that have been developed which can be divided into passive wearables (either wrist worn or attached to lower back or legs), or active devices (either on a custom made electronic device, or available as an App to download onto an individual's own smartphone/tablet).^59^ The passive technologies can be used to collect very large amounts of data to inform on an individual's routine performance such as multiple aspects of mobility, their hours spent asleep, or the severity/frequency of tremor, bradykinesia, or dyskinesia.^60^ The active devices instead explore the capacity of an individual to complete tasks e.g., finger tapping/arm movements, or can even be adapted to collect data on speech or cognition. Outside of clinical trial settings, these devices are also increasingly being proposed for remote self assessment/management.^61^ The usefulness of any of these technologies depends not only on the quality of the accelerometry sensors or GPS systems contained within the devices, but also very much on the algorithms that have been created to extract the data and translate it into quantitative metrics that can be compared within and between patients.^62^

These technologies are of great appeal in increasing the objectivity of assessment of PD progression, as evaluated in an individual's real world environment, however potential confounds exist such as the influence of weather, seasons, changes in work or domestic commitments that may have profound influence on the extracted metrics e.g., Step counts, and need to be carefully incorporated as covariates when comparing changes over time.

The demonstration that a candidate intervention has an impact on a digital metric such as number of walking bouts, or stride length, may be extremely useful information, but taken on their own are unlikely to allow the intervention to pass the threshold for regulatory approval. What is needed is confirmation that changes in digital measures can reliably predict a measure of clinical progression that is relevant to patients. Hence embedding digital measures into a trial that contemporaneously collects Patient reported outcomes is of vital importance to validate these tools.

There are also multiple teams trying to develop methods of accurately imaging the extent of alpha synuclein pathology within the brain.^63–65^ Confirmation of the specificity of the ligands for alpha synuclein, (rather than amyloid or tau or other proteinopathy) will require extensive validation work, however early inclusion as exploratory endpoints into clinical trials will be of enormous value to this end. The use of PET ligands to quantify the extent of PD pathology may be of major importance for trials and academic insights, but are inevitably less available for the use in clinical practice. For this reason the use of quantitative MRI techniques to assess changes in the structure of e.g., the substantia nigra over time are also being pursued.^66,67^

Similarly the development of a wet biomarker that accurately reflects the quantitative stage or severity of PD and that is relevant to the majority of PD patients, and that is accessible with a minimally invasive approach is a major need.^68^ One such avenue is the development of a quantitative version of the alpha synuclein SAA,^69^ but broadening the biomarker analyses in trial settings will allow identification of other critical convergent mechanisms of disease and help in their validation.

The potential of using digital measures, quantitative imaging or a quantitative SAA in capturing change/progression among people with prodromal PD, (before an easily recognisable clinical syndrome has developed), has also been considered^70^ and certainly hold promise to allow shorter, smaller proof of concept in prevention trials such as P2P, as well as in the context of MAMS trials such as EJS ACT-PD.

## Conclusion

There has been a major shift in trial design in the search for disease modifying approaches in PD. Increasing the efficiency with which we can assess the most encouraging compounds, alongside careful consideration of which patients to target (bearing in mind patient heterogeneity, disease stage and the importance of inclusivity) has led to the development of several major new platform trials. The purpose of these trials is of course to accelerate the identification and licensing of effective disease modifying treatments for PD, but also to allow efficient identification of those agents which are ineffective and can be rapidly deprioritised.

An important parallel process that is being incorporated into these trials is the development of improved ways of measuring disease progression, using a combination of wet biomarkers, imaging biomarkers and digital wearables, and their relationship to patient relevant milestones of disease progression. At the current time, long term follow up is essential to confirm clinically relevant effects and reduce the risk of inappropriately concluding that an intervention is ineffective because of inadequate follow up duration. By assisting in the development and assessment of improved biomarkers, we can expect these platform trials to continue to evolve in their design to become even more time and cost efficient.

The success of these trials will rely on the attention to detail in their designs. These details have been carefully developed and refined with input from people with PD, charities and funders that have been staunch supporters of their creation. EJS ACT-PD has benefited greatly from the expertise of the MRC CTU, who have a long track record in successful delivery of MAMS platform trials, and is a member of the ACORD initiative, a collaboration of investigators leading on trials in PD, Multiple sclerosis, Motor Neuron disease and Alzheimer's disease. There is also close communication and international co-ordination between PD platform trials to ensure these platform approaches are complementary and will inform on treatment selection priorities for each other.

The establishment of a co-ordinated infrastructure of PD disease modifying trials across the globe^71^ represents a significant step forward towards identification of effective treatments. Regular communication between teams leading these trials also allows for coordination of data management for example using common data elements will facilitate cross-trial analyses, and AI/ML approaches in the analysis of multi-modal datasets.^72^ Ultimately there may need to be individual tailoring of treatment selection to specific patients based on preclinical responsiveness e.g., using individual patient derived stem cells,^73^ and there may be a need to test combinations of medications rather than expectation that a single agent will be sufficiently effective alone. Efficient communication and planning within and across trials will be key to the success of this.

## References

[1] Postuma RB Berg D Stern M , et al. MDS Clinical diagnostic criteria for Parkinson's disease. Mov Disord 2015; 30: 1591–1601. 26474316 10.1002/mds.26424

[2] Standke HG Kraus A . Seed amplification and RT-QuIC assays to investigate protein seed structures and strains. Cell Tissue Res 2023; 392: 323–335. 35258712 10.1007/s00441-022-03595-z

[3] Okuzumi A Hatano T Matsumoto G , et al. Propagative alpha-synuclein seeds as serum biomarkers for synucleinopathies. Nat Med 2023; 29: 1448–1455. 37248302 10.1038/s41591-023-02358-9 PMC10287557

[4] Yan S Jiang C Janzen A , et al. Neuronally derived extracellular vesicle alpha-Synuclein as a Serum biomarker for individuals at risk of developing Parkinson disease. JAMA Neurol 2024; 81: 59–68. 38048087 10.1001/jamaneurol.2023.4398 PMC10696516

[5] Siderowf A Concha-Marambio L Lafontant DE , et al. Assessment of heterogeneity among participants in the Parkinson's progression markers initiative cohort using alpha-synuclein seed amplification: a cross-sectional study. Lancet Neurol 2023; 22: 407–417. 37059509 10.1016/S1474-4422(23)00109-6 PMC10627170

[6] Simuni T Chahine LM Poston K , et al. A biological definition of neuronal alpha-synuclein disease: towards an integrated staging system for research. Lancet Neurol 2024; 23: 178–190. 38267190 10.1016/S1474-4422(23)00405-2

[7] Espay AJ Cardoso F Frucht SJ , et al. Refutation of the alphaSyn-SAA-based staging for Parkinson's progression (neuronal alpha-Synuclein disease-integrated staging system [NSD-ISS]). Mov Disord 2025; 40: 1744–1745. 40579848 10.1002/mds.30269 PMC12371609

[8] Kim A Martinez-Valbuena I Keith JL , et al. Misfolded alpha-Synuclein seeding is detected in suspected LRRK2-Parkinson's disease without immunohistochemically detectable alpha-Synuclein pathology. Mov Disord 2024; 39: 218–220. 37986700 10.1002/mds.29665

[9] Sekiya H Franke L Hashimoto Y , et al. Widespread distribution of alpha-synuclein oligomers in LRRK2-related Parkinson's disease. Acta Neuropathol 2025; 149: 42. 40314842 10.1007/s00401-025-02872-9 PMC12395563

[10] Foltynie T Brayne C Barker RA . The heterogeneity of idiopathic Parkinson's disease. J Neurol 2002; 249: 138–145. 11985378 10.1007/pl00007856

[11] Dulski J Uitti RJ Ross OA , et al. Genetic architecture of Parkinson's disease subtypes – review of the literature. Front Aging Neurosci 2022; 14: 1023574. 36337703 10.3389/fnagi.2022.1023574 PMC9632166

[12] Dulski J Heckman MG White LJ , et al. Distinct biological underpinnings of Parkinson's disease motor subtypes: single-center study on pathway-stratified polygenic risk scores. Parkinsonism Relat Disord 2025; 137: 107901. 40483897 10.1016/j.parkreldis.2025.107901

[13] Albrecht F Poulakis K Freidle M , et al. Unraveling Parkinson's disease heterogeneity using subtypes based on multimodal data. Parkinsonism Relat Disord 2022; 102: 19–29. 35932584 10.1016/j.parkreldis.2022.07.014

[14] Mestre TA Fereshtehnejad SM Berg D , et al. Parkinson's disease subtypes: critical appraisal and recommendations. J Parkinsons Dis 2021; 11: 395–404. 33682731 10.3233/JPD-202472 PMC8150501

[15] Abeliovich A Hefti F Sevigny J . Gene therapy for Parkinson's disease associated with GBA1 mutations. J Parkinsons Dis 2021; 11: S183–S1S8. 10.3233/JPD-212739 PMC8543272 34151863

[16] Mata I Salles P Cornejo-Olivas M , et al. LRRK2: genetic mechanisms vs genetic subtypes. Handb Clin Neurol 2023; 193: 133–154. 36803807 10.1016/B978-0-323-85555-6.00018-7

[17] Lubben N Brynildsen JK Webb CM , et al. LRRK2 Kinase inhibition reverses G2019S mutation-dependent effects on tau pathology progression. Transl Neurodegener 2024; 13: 13. 38438877 10.1186/s40035-024-00403-2 PMC10910783

[18] Poewe W . The natural history of Parkinson's disease. J Neurol 2006; 253: VII2–VII6. 10.1007/s00415-006-7002-7 17131223

[19] West AB Schwarzschild MA . LRRK2-targeting therapies march through the valley of death. Mov Disord 2023; 38: 361–365. 36942368 10.1002/mds.29343 PMC11076002

[20] Jiang Z Huang H Chen Y , et al. The role of the immune system in Parkinson's disease pathogenesis: a focus on Th17 cells – A systematic review and meta-analysis. J Neuroimmunol 2025; 398: 578484. 39577101 10.1016/j.jneuroim.2024.578484

[21] Raheel K Deegan G Di Giulio I , et al. Sex differences in alpha-synucleinopathies: a systematic review. Front Neurol 2023; 14: 1204104. 37545736 10.3389/fneur.2023.1204104 PMC10398394

[22] Athauda D Foltynie T . Insulin resistance and Parkinson's disease: a new target for disease modification? Prog Neurobiol 2016; 145-146: 98–120. 27713036 10.1016/j.pneurobio.2016.10.001

[23] Dorsey ER Bloem BR . Parkinson's disease is predominantly an environmental disease. J Parkinsons Dis 2024; 14: 451–465. 38217613 10.3233/JPD-230357 PMC11091623

[24] Djamshidian A Lees AJ . Can stress trigger Parkinson's disease? J Neurol Neurosurg Psychiatry 2014; 85: 878–881. 24259593 10.1136/jnnp-2013-305911

[25] Tan MMX Lawton MA Pollard MI , et al. Genome-wide determinants of mortality and motor progression in Parkinson's disease. NPJ Parkinsons Dis 2024; 10: 113. 38849413 10.1038/s41531-024-00729-8 PMC11161485

[26] Vijiaratnam N Simuni T Bandmann O , et al. Progress towards therapies for disease modification in Parkinson's disease. Lancet Neurol 2021; 20: 559–572. 34146514 10.1016/S1474-4422(21)00061-2

[27] Gonzalez-Robles C Athauda D Barber TR , et al. Treatment selection and prioritization for the EJS ACT-PD MAMS trial platform. Mov Disord 2025; 40: 1307–1317. 40249275 10.1002/mds.30190 PMC12273612

[28] Brauer R Wei L Ma T , et al. Diabetes medications and risk of Parkinson's disease: a cohort study of patients with diabetes. Brain 2020; 143: 3067–3076. 33011770 10.1093/brain/awaa262 PMC7794498

[29] Gubellini P Kachidian P . Animal models of Parkinson's disease: an updated overview. Rev Neurol (Paris) 2015; 171: 750–761. 26343921 10.1016/j.neurol.2015.07.011

[30] Polinski NK . A summary of phenotypes observed in the in vivo rodent alpha-Synuclein preformed fibril model. J Parkinsons Dis 2021; 11: 1555–1567. 34486988 10.3233/JPD-212847 PMC8609716

[31] Beckstead MJ Howell RD . Progressive parkinsonism due to mitochondrial impairment: lessons from the MitoPark mouse model. Exp Neurol 2021; 341: 113707. 33753138 10.1016/j.expneurol.2021.113707 PMC8169575

[32] Choi ML Chappard A Singh BP , et al. Pathological structural conversion of alpha-synuclein at the mitochondria induces neuronal toxicity. Nat Neurosci 2022; 25: 1134–1148. 36042314 10.1038/s41593-022-01140-3 PMC9448679

[33] Fang TZ Sun Y Pearce AC , et al. Knockout or inhibition of USP30 protects dopaminergic neurons in a Parkinson's disease mouse model. Nat Commun 2023; 14: 7295. 37957154 10.1038/s41467-023-42876-1 PMC10643470

[34] https://www.helse-bergen.no/en/neuro-sysmed-english/clinical-studies-at-neuro-sysmed/parkinsons-clinical-studies/sleipnir/ .

[35] Wyse RK Isaacs T Barker RA , et al. Twelve years of drug prioritization to help accelerate disease modification trials in Parkinson's disease: the international linked clinical trials initiative. J Parkinsons Dis 2024; 14: 657–666. 38578902 10.3233/JPD-230363 PMC11191436

[36] Ravina BM Fagan SC Hart RG , et al. Neuroprotective agents for clinical trials in Parkinson's disease: a systematic assessment. Neurology 2003; 60: 1234–1240. 12707423 10.1212/01.wnl.0000058760.13152.1a

[37] Love SB Cafferty F Snowdon C , et al. Practical guidance for running late-phase platform protocols for clinical trials: lessons from experienced UK clinical trials units. Trials 2022; 23: 757. 36068599 10.1186/s13063-022-06680-4 PMC9449272

[38] Fabbri M Rascol O Foltynie T , et al. Advantages and challenges of platform trials for disease modifying therapies in Parkinson's disease. Mov Disord 2024; 39: 1468–1477. 38925541 10.1002/mds.29899

[39] Lewis SJ Sue CM Cooper A , et al. Future is now: an Australasian perspective on disease-modifying trials in Parkinson's and prodromal disease. BMJ Neurol Open 2025; 7: e001070. 10.1136/bmjno-2025-001070 PMC11931937 40129903

[40] James ND Sydes MR Clarke NW , et al. Addition of docetaxel, zoledronic acid, or both to first-line long-term hormone therapy in prostate cancer (STAMPEDE): survival results from an adaptive, multiarm, multistage, platform randomised controlled trial. Lancet 2016; 387: 1163–1177. 26719232 10.1016/S0140-6736(15)01037-5 PMC4800035

[41] Pal S Chataway J Swingler R , et al. Safety and efficacy of memantine and trazodone versus placebo for motor neuron disease (MND SMART): stage two interim analysis from the first cycle of a phase 3, multiarm, multistage, randomised, adaptive platform trial. Lancet Neurol 2024; 23: 1097–1107. 39307154 10.1016/S1474-4422(24)00326-0

[42] Gray E Amjad A Robertson J , et al. Enhancing involvement of people with multiple sclerosis in clinical trial design. Mult Scler 2023; 29: 1162–1173. 37555494 10.1177/13524585231189678 PMC10413782

[43] Foltynie T Gandhi S Gonzalez-Robles C , et al. Towards a multi-arm multi-stage platform trial of disease modifying approaches in Parkinson's disease. Brain 2023; 146: 2717–2722. 36856727 10.1093/brain/awad063 PMC10316775

[44] Stephenson D Ollivier C Brinton R , et al. Can innovative trial designs in orphan diseases drive advancement of treatments for common neurological diseases? Clin Pharmacol Ther 2022; 111: 799–806. 35034352 10.1002/cpt.2528 PMC9305159

[45] Zeissler ML Li V Parmar MKB , et al. Is it possible to conduct a multi-arm multi-stage platform trial in Parkinson's disease: lessons learned from other neurodegenerative disorders and cancer. J Parkinsons Dis 2020; 10: 413–428. 32116263 10.3233/JPD-191856 PMC7242843

[46] Zeissler ML Bakshi N Bartlett M , et al. Patient and public involvement and engagement in the development of a platform clinical trial for Parkinson's disease: an evaluation protocol. J Parkinsons Dis 2024; 14: 809–821. 38701161 10.3233/JPD-230444 PMC11191543

[47] Gonzalez-Robles C Bartlett M Burnell M , et al. Embedding patient input in outcome measures for long-term disease-modifying Parkinson disease trials. Mov Disord 2024; 39: 433–438. 38140767 10.1002/mds.29691

[48] Petty R Agarwal V Allison J , et al. Improving recruitment and retention of people with Parkinson's disease to clinical studies: a scoping review. J Parkinsons Dis 2025; 15: 6–18. 39973478 10.1177/1877718X241291986 PMC13347406

[49] Gilson C Chowdhury S Parmar MKB , et al. Incorporating biomarker stratification into STAMPEDE: an adaptive multi-arm, multi-stage trial platform. Clin Oncol (R Coll Radiol) 2017; 29: 778–786. 29079227 10.1016/j.clon.2017.10.004 PMC5710986

[50] Gonzalez-Robles C Weil RS van Wamelen D , et al. Outcome measures for disease-modifying trials in Parkinson's disease: consensus paper by the EJS ACT-PD multi-arm multi-stage trial initiative. J Parkinsons Dis 2023; 13: 1011–1033. 37545260 10.3233/JPD-230051 PMC10578294

[51] Goetz CG Tilley BC Shaftman SR , et al. Movement disorder society-sponsored revision of the unified Parkinson's disease rating scale (MDS-UPDRS): scale presentation and clinimetric testing results. Mov Disord 2008; 23: 2129–2170. 19025984 10.1002/mds.22340

[52] Hill DL Carroll C Belfiore-Oshan R , et al. Aligning with regulatory agencies for the use of digital health technologies in drug development: a case study from Parkinson's disease. Front Digit Health 2025; 7: 1415202. 41208852 10.3389/fdgth.2025.1415202 PMC12589013

[53] Tosin MHS Simuni T Stebbins GT , et al. Tracking emergence of new motor and non-motor symptoms using the MDS-UPDRS: a novel outcome measure for early Parkinson's disease? J Parkinsons Dis 2022; 12: 1345–1351. 35466955 10.3233/JPD-223170 PMC9198734

[54] Brumm MC Siderowf A Simuni T , et al. Parkinson's progression markers initiative: a milestone-based strategy to monitor Parkinson's disease progression. J Parkinsons Dis 2023; 13: 899–916. 37458046 10.3233/JPD-223433 PMC10578214

[55] Quattrone A Barbagallo G Cerasa A , et al. Neurobiology of placebo effect in Parkinson's disease: what we have learned and where we are going. Mov Disord 2018; 33: 1213–1227. 30230624 10.1002/mds.27438

[56] Lidstone SC . Great expectations: the placebo effect in Parkinson's disease. Handb Exp Pharmacol 2014; 225: 139–147. 25304530 10.1007/978-3-662-44519-8_8

[57] Haji S Sako W Murakami N , et al. Factors associated with a placebo effect in Parkinson's disease in clinical trials: a meta-analysis. J Neurol 2024; 271: 5825–5837. 38955829 10.1007/s00415-024-12529-4

[58] Lee S Walker JR Jakul L , et al. Does elimination of placebo responders in a placebo run-in increase the treatment effect in randomized clinical trials? A meta-analytic evaluation. Depress Anxiety 2004; 19: 10–19. 14978780 10.1002/da.10134

[59] Rabano-Suarez P Del Campo N Benatru I , et al. Digital outcomes as biomarkers of disease progression in early Parkinson's disease: a systematic review. Mov Disord 2025; 40: 184–203. 39613480 10.1002/mds.30056 PMC11832816

[60] Del Din S Kirk C Yarnall AJ , et al. Body-worn sensors for remote monitoring of Parkinson's disease motor symptoms: vision, state of the art, and challenges ahead. J Parkinsons Dis 2021; 11: S35–S47. 10.3233/JPD-202471 PMC8385520 33523020

[61] Frohlich H Bontridder N Petrovska-Delacreta D , et al. Leveraging the potential of digital technology for better individualized treatment of Parkinson's disease. Front Neurol 2022; 13: 788427. 35295840 10.3389/fneur.2022.788427 PMC8918525

[62] Chandrabhatla AS Pomeraniec IJ Ksendzovsky A . Co-evolution of machine learning and digital technologies to improve monitoring of Parkinson's disease motor symptoms. NPJ Digit Med 2022; 5: 32. 35304579 10.1038/s41746-022-00568-y PMC8933519

[63] Newswire P. XingImaging in partnership with SynuSight was awarded a grant from The Michael J. Fox Foundation to support development of the α-synuclein PET tracer 18F-FD4 for Parkinson's disease. 2025. https://www.prnewswire.com/news-releases/xingimaging-in-partnership-with-synusight-was-awarded-a-grant-from-the-michael-j-fox-foundation-to-support-development-of-the–synuclein-pet-tracer-18f-fd4-for-parkinsons-disease-302553154.html .

[64] Endo H Ono M Takado Y , et al. Imaging alpha-synuclein pathologies in animal models and patients with Parkinson's and related diseases. Neuron 2024; 112: 2540–57.e8. 38843838 10.1016/j.neuron.2024.05.006

[65] Smith R Capotosti F Schain M , et al. The alpha-synuclein PET tracer [18F] ACI-12589 distinguishes multiple system atrophy from other neurodegenerative diseases. Nat Commun 2023; 14: 6750. 37891183 10.1038/s41467-023-42305-3 PMC10611796

[66] Yan Y Zhang M Ren W , et al. Neuromelanin-sensitive magnetic resonance imaging: possibilities and promises as an imaging biomarker for Parkinson's disease. Eur J Neurosci 2024; 59: 2616–2627. 38441250 10.1111/ejn.16296

[67] Lambert C Chowdhury R Fitzgerald TH , et al. Characterizing aging in the human brainstem using quantitative multimodal MRI analysis. Front Hum Neurosci 2013; 7: 462. 23970860 10.3389/fnhum.2013.00462 PMC3747448

[68] Vijiaratnam N Foltynie T . How should we be using biomarkers in trials of disease modification in Parkinson's disease? Brain 2023; 146: 4845–4869. 37536279 10.1093/brain/awad265 PMC10690028

[69] Abdi IY Sudhakaran IP Ghanem SS , et al. Quantitative measurements of alpha-synuclein seeds in CSF inform diagnosis of synucleinopathies. J Parkinsons Dis 2025 Dec; 15: 1412–1430. 41042913 10.1177/1877718X251379292 PMC13347542

[70] Mirelman A Siderowf A Chahine L . Outcome assessment in Parkinson disease prevention trials: utility of clinical and digital measures. Neurology 2022; 99: 52–60. 35970590 10.1212/WNL.0000000000200236

[71] Parkinson’s C. 2025. https://cureparkinsons.org.uk/2025/11/launch-of-a-global-alliance-for-parkinsons-platforms-gapp-bringing-together-international-leaders-of-parkinsons-platform-trials/ .

[72] Huang G Li R Bai Q , et al. Multimodal learning of clinically accessible tests to aid diagnosis of neurodegenerative disorders: a scoping review. Health Inf Sci Syst 2023; 11: 32. 37489153 10.1007/s13755-023-00231-0 PMC10363100

[73] D'Sa K Evans JR Virdi GS , et al. Prediction of mechanistic subtypes of Parkinson's using patient-derived stem cell models. Nat Mach Intell 2023; 5: 933–946. 37615030 10.1038/s42256-023-00702-9 PMC10442231

